# *Mycobacterium bovis* Wild-Type BCG or Recombinant BCG Secreting Murine IL-18 (rBCG/IL-18) Strains in Driving Immune Responses in Immunocompetent or Immunosuppressed Mice

**DOI:** 10.3390/vaccines10040615

**Published:** 2022-04-14

**Authors:** Marek Fol, Marcin Włodarczyk, Magdalena Kowalewicz-Kulbat, Magdalena Druszczyńska, Krzysztof T. Krawczyk, Sebastian Wawrocki, Wiesława Rudnicka, Magdalena Chmiela

**Affiliations:** Department of Immunology and Infectious Biology, Faculty of Biology and Environmental Protection, University of Lodz, Banacha 12/16, 90-237 Lodz, Poland; marcin.wlodarczyk@biol.uni.lodz.pl (M.W.); magdalena.kowalewicz@biol.uni.lodz.pl (M.K.-K.); magdalena.druszczynska@biol.uni.lodz.pl (M.D.); krzysztof.krawczyk@biol.uni.lodz.pl (K.T.K.); sebastian.wawrocki@biol.uni.lodz.pl (S.W.); wieslawa.rudnicka@biol.uni.lodz.pl (W.R.); magdalena.chmiela@biol.uni.lodz.pl (M.C.)

**Keywords:** BCG, IL-18, immunosuppression, cyclophosphamide, immunity, C3H, C57BL/6

## Abstract

*Mycobacterium tuberculosis* infections remain a global health problem in immunosuppressed patients. The effectiveness of BCG (Bacillus Calmette–Guérin), an anti-tuberculosis vaccine, is unsatisfactory. Finding a new vaccine candidate is a priority. We compared numerous immune markers in BCG-susceptible C57BL/6 and BCG-resistant C3H mice who had been injected with 0.9% NaCl (control) or with wild-type BCG or recombinant BCG secreting interleukin (IL)-18 (rBCG/IL-18) and in immunized mice who were immunocompromised with cyclophosphamide (CTX). The inoculation of rBCG/IL-18 in immunocompetent mice increased the percentage of bone marrow myeloblasts and promyelocytes, which were further elevated in the rBCG/IL-18/CTX-treated mice: C57BL/6 mice—3.0% and 11.4% (control) vs. 18.6% and 42.4%, respectively; C3H mice—1.1% and 7.7% (control) vs. 18.4% and 44.9%, respectively, *p* < 0.05. The bone marrow cells showed an increased mean fluorescence index (MFI) in the CD34 adhesion molecules: C57BL/6 mice—4.0 × 10^3^ (control) vs. 6.2 × 10^3^; C3H mice—4.0 × 10^3^ (control) vs. 8.0 × 10^3^, *p* < 0.05. Even in the CTX-treated mice, the rBCG/IL-18 mobilized macrophages for phagocytosis, C57BL/6 mice—4% (control) vs. 8%; C3H mice—2% (control) vs. 6%, and in immunocompetent mice, C57BL/6 induced the spleen homing of effector memory CD4^+^ and CD8^+^ T cells (T_EM_), 15% (control) vs. 28% and 8% (control) vs. 22%, respectively, *p* < 0.05. In conclusion, rBCG/IL-18 effectively induced selected immune determinants that were maintained even in immunocompromised mice.

## 1. Introduction

Tuberculosis (TB), a disease caused by *Mycobacterium tuberculosis* (M.tb), remains a global clinical and social problem. Annually, 7–10 million new cases occur, approximately 2 million people die from TB worldwide, and those who are infected constitute a reservoir of tubercle bacilli [1,2]. In the latent (dormant) state, M.tb exists in a quiescent state, constrained by host immune responses within granulomas or other protected sites. Latent M.tb infection has a 5–15% risk of progressing to active TB due to waning immunity [3]. The risk of TB infection or the reactivation of infection increases dramatically in people undergoing immunosuppressive treatment. The only effective way to protect immunocompromised patients with latent M.tb infection from the development of active TB is prophylactically administered antitubercular therapy [4]; however, such treatments cause side effects, including liver injury, peripheral neuropathy, dermatitis, and metabolic acidosis, making patients worse off [5]. Another possibility is prophylactic immunization using the Bacillus Calmette–Guérin (BCG) vaccine—a live attenuated vaccine containing *Mycobacterium bovis* bacilli (*M. bovis* BCG), which is so far the only available vaccine against TB [6]; however, as the risk of latent M.tb infection was not found to be lower in BCG-vaccinated individuals, they still remain a potent source of new M.tb transmissions. Thus, searching for a vaccine that is more effective than BCG for enhancing antitubercular immunity to serve as a tool for preventing TB and the reactivation of latent infection in immunocompromised patients is an urgent challenge. 

*M. bovis* BCG is an attractive candidate for a recombinant vaccine with remarkable adjuvant activity and that is capable of activating both innate and adaptive immunity [7,8]. Recombinant BCG strains can express foreign antigens on the surface and in the cytoplasm or can secrete important immunomodulatory mediators to the environment. Mouse models that are susceptible or resistant to BCG can help us to understand the potential immune mechanisms driven by wild-type or recombinant BCG strains in both immunocompetent and immunocompromised subjects. In our study, we used the recombinant *M. bovis* BCG strain secreting murine interleukin (IL)-18 (rBCG/IL-18) as a prototype of a modified BCG anti-TB vaccine that could potentially be more effective than wild-type BCG in driving an immune response that is able to prevent the development of active TB. The rBCG/IL-18 strain was constructed at the Institut Pasteur de Lille, France, and showed enhanced immunomodulatory properties, resulting in the development of the type 1 T helper (h) lymphocyte response in mouse studies [9,10]. IL-18, originally discovered as an interferon (IFN)-gamma (γ)-inducing factor, is regarded as a potent regulator of both innate and acquired immune responses [11,12]. Along with IL-12 or IL-15, IL-18 induces the activity of natural killer (NK) cells and directs immunity towards the Th1 cell response, that is characterized by profound IFN-γ production, which is a key factor for increasing the antibacterial activity of macrophages [12,13,14,15,16]. The IL-18-driven increase in IFN-γ production is accompanied by enhanced T cell proliferation and the production of other cytokines and growth factors such as IFN-γ, tumor necrosis factor (TNF)-α, granulocyte-macrophage colony-stimulating factor (GM-CSF), IL-14, IL-5, and IL-13 by T helper (CD4^+^) lymphocytes as well as the activation of cytotoxic T (CD8^+^) lymphocytes. It was shown that intraperitoneal IL-18 injection in immunocompromised mice infected with pathogens such as *Escherichia coli*, *Listeria monocytogenes*, *Staphylococcus aureus*, and *Cryptococcus neoformans*, enhanced both the Th1 and Th2 responses, humoral immunity, and antibacterial activity of neutrophils [17]. 

Previously, rBCG/IL-18 had never been tested in an immunosuppression model. Taking into account the diminished activity of the immune system due to therapeutic immunosuppression, we developed two mouse models, including mice strains that are sensitive or resistant to *M.bovis* BCG, to compare the immune status of immunocompetent animals that have been exposed to wild-type BCG or rBCG/IL-18 with the immune status of mice who have been exposed to these BCG formulations followed by immunosuppression. We used two strains of mice that differed in their BCG susceptibility, BCG-susceptible (C57BL/6 Bcgs) and BCG-resistant C3H (Bcgr), to see whether genetically determined defense mechanisms affect the immune responses to wild-type or rBCG/IL-18 bacilli in mice who have been treated and who have not been treated with cyclophosphamide (CTX). To some extent, this double mouse model can mimic the situation in humans in whom their genetic background may influence the outcome of mycobacterial infection. The differences attributed to susceptibility or resistance to BCG in mice were related to granuloma formation in the liver and spleen, delayed-type hypersensitivity, and resistance to the challenge with homologous (BCG) and heterologous (*Listeria monocytogenes*) pathogens [18,19].

The aim of this study was to show whether rBCG/IL-18 induced selective immune response determinants compared to those induced by wild-type BCG and whether those immune mechanisms could be restored in mice who had been immunized with recombinant BCG and then immunocompromised with CTX, potentially providing better protection against mycobacteria. We selected CTX as an immunosuppressive agent because it is used for the treatment of autoimmune diseases, in eradication therapy for malignant hematopoietic cells, and for the prevention of transplant rejection or graft-vs-host complications in humans [20]. CTX has a profound effect on immune responses that are mediated by B and T cells, including regulatory T lymphocytes. It was revealed that in humans, CTX decreased the systemic level of Th1 cytokines (IFN-γ and IL-12) and increased the secretion of Th2 cytokines such as IL-4 and IL-10 [21]. CTX’s strong immunomodulatory activity in tumor cell killing and in immune memory induction, which enabled tumor surveillance, was evident when CTX was used at low doses [22]. The optimal CTX dosage and delivery time for the induction of immunosuppression in mice in our models was experimentally established in our previous study [23].

In immunocompetent mice, rBCG/IL-18 inoculation vs. 0.9% NaCl (control) resulted in an increased percentage of bone marrow myeloblasts and promyelocytes, which was further elevated in immunized mice who had been treated with CTX (rBCG/IL-18/CTX) while the number of mature granulocytes was lower. CD34 adhesion molecule expression was enhanced on bone marrow cells in immunocompetent mice who had been inoculated with rBCG/IL-18 and particularly in rBCG/IL-18/CTX animals. The rBCG/IL-18 vaccination mobilized macrophages to undergo the phagocytosis of mycobacteria, even in immunized mice who had been treated with CTX, and in C57BL/6 mice, vaccination resulted in the spleen homing of effector memory CD4^+^ and CD8^+^ T cells (T_EM_). This study showed that rBCG/IL-18 effectively induced selected immune determinants, which were even able to be maintained in immunocompromised mice. Nonetheless, further investigations are required to determine the beneficial effects of rBCG/IL-18 in driving anti-mycobacterial immune responses in practice.

## 2. Materials and Methods

### 2.1. Animals

Sibling groups of C57BL/6 (Bcgs) and C3H (Bcgr) mice were purchased from Charles River Laboratories (Germany). They were matched in the breeding animal facility of the Institute of Microbiology, Biotechnology and Immunology, University of Lodz (Poland) to produce subsequent inbred generations. All animals were housed in ventilated cages in an environmentally controlled room (20–25 °C, 50 ± 10% humidity, 12 h dark/light cycles) and were provided with pelleted feed for rodents and water ad libitum. Eight-to-ten-week-old male mice were used for all experiments and were then euthanized by intraperitoneal (i.p.) injection with Morbital (a formulation of sodium pentobarbital: 133.3 mg/mL and pentobarbital: 26.7 mg/mL; Biowet, Puławy, Poland) at a dose of 2 mL/kg body weight in order to collect biological samples for assessment. The protocol was approved by the Local Ethics Committee for Animal Experimentation in Lodz (decision No. 33/ŁB717/2014).

### 2.2. Bacteria Growth Conditions

*Mycobacterium bovis* wild-type BCG (substrain 1173P2, World Health Organization, Stockholm, Sweden) and the recombinant *M. bovis* BCG strain secreting murine interleukin (IL)-18 (rBCG/IL-18), a gift from Dr. Camille Locht (Center for Infection and Immunity of Lille, Institut Pasteur de Lille, Université de Lille, Lille, France) and Dr. Franck Biet (Institut National de Recherche pour l’Agriculture, l’Alimentation et l’Environnement (INRAE), Université de Tours, Nouzilly, France), were cultivated in Middlebrook 7H9 broth (Difco, Detroit, MI, USA) supplemented with oleic acid-albumin-dextrose-catalase enrichment (OADC) (Life Technologies, Gaithersburg, MD, USA), 0.2% glycerol (POCH, Gliwice, Poland), and 0.05% Tween 80 (Sigma-Aldrich, Steinheim, Germany). The rBCG/IL-18 growth medium was additionally supplemented with HgCl_2_ (10 μg/mL) [9]. Mid-log-phase cultures were harvested, aliquoted, and frozen at −80°C. After thawing, the number of viable cells was determined by plating serial dilutions of the cultures on Middlebrook 7H10 agar containing 10% OADC (and mercuric chloride for rBCG/IL-18) followed by incubation at 37 °C for 3 weeks. To ensure a clump-free suspension of mycobacteria, 1 ml of the suspension was drawn through a nonpyrogenic needle (Microlance, 0.45 × 16, 26 G × 5/8′; BD, Drogheda, Ireland). Our laboratory facility, where the experiments were conducted, has governmental approval to work with genetically modified microorganisms (GMM).

### 2.3. Infection and Immunosuppression of Mice

The following groups of mice were used: immunocompetent animals, which were either intracutaneously (i.c.) injected with 0.9% NaCl or i.c. immunized with the wild-type BCG or rBCG/IL-18 and i.p. injected with 0.9% NaCl for 7 consecutive days, 6 weeks later (groups: NaCl/NaCl, wild-type BCG/NaCl, rBCG/IL-18/NaCl, respectively), and immunocompromised mice, which were injected with 0.9% NaCl or immunized with the wild-type BCG or rBCG/IL-18 (as above) and then i.p. injected with CTX for 7 days (groups: NaCl/CTX, wild-type BCG/CTX, rBCG/IL-18/CTX, respectively). 

Mycobacteria were suspended in 0.9% NaCl and administered i.c. using a Myjector (29 G, 0.33 × 12, Terumo, Leuven, Belgium) at two spots into the loose skin over the interscapular area (30μL/spot containing approximately 5000 mycobacteria/spot). At the time of immunization, the animals were transiently anesthetized (15–30 min) with a mixture of Ketamine 10% (ketamine hydrochloride, 115.34 mg/mL) and Sedazin (xylazine hydrochloride, 20 mg/mL) (both from Biowet, Puławy, Poland), which was administered i.p. The control mice received an equivalent volume of 0.9% NaCl. Six weeks later, the mice were treated i.p. with cyclophosphamide (CTX, Endoxan; Baxter Oncology GmbH, Halle, Germany) at a dose of 50 µg/g b.w. for a subsequent seven days, as previously described [23]. The control mice were injected with NaCl (0.9%). A total of 24 h after the last dose of CTX or saline administration, the animals were euthanized, and biological samples were collected for further examination. We used four animals per each group, in accordance with the principle of reducing the number of animals tested. Each experiment was repeated three times. The number of animals per group was sufficient for statistical analysis. Immunosuppression status was confirmed as previously indicated and was determined based on the composition of the peripheral blood leukocytes (leukocyte index) and selected humoral parameters, including the total serum protein content, and total levels of albumins and alpha-, beta-, and gamma-globulins. Moreover, the proliferating activity of the splenocytes in response to phytohemagglutinin was examined via the [^3^H]-thymidine incorporation assay [23]. 

### 2.4. Isolation of Bone Marrow Cells and Cytospin Preparation

The femurs were excised, and a 25-gauge needle attached to a 1 mL insulin syringe filled with ice-cold RPMI medium was used to flush the bone marrow cells out [24]. Next, the cell suspension was centrifuged (+4 °C, 350× *g*, 10 min), and the pellet was suspended in 1 mL of phosphate-buffered saline (PBS) to determine the cell density. Finally, 3 × 10^5^ cells in 300 µL of PBS with 1% bovine serum albumin (BSA) were used to prepare a single cytospin using the cytology set (MPW Med. Instruments, Warszawa, Poland). The cells were fixed in 4% paraformaldehyde (PFA) for 30 min, washed in PBS for 5 min, air-dried at room temperature, and stored until use for Pappenheim staining [25] or for the immunofluorescent staining (as indicated below) of the cluster differentiation (CD) surface molecules CD34 and CD117, which play a role in the adhesion and migration (CD34) as well as in the proliferation, differentiation, and apoptosis (CD117) of hematopoietic stem cells (HSCs) [26,27,28,29,30,31,32]. Cellular composition analysis, including the identification of individual cell types, was performed by two independent histopathologists in a specialistic histopathology unit—Institut für Tierpathologie, Berlin, Germany. The percentages of lymphocytes, monocytes, and granulocytes were assessed in all of the experimental variants. Additionally, myeloblasts, promyelocytes, myelocytes, and metamyelocytes were recognized in accordance with the guidelines contained in “*Mouse Hematology: A Laboratory Manual*” [33] and based on the nucleus to cytoplasm ratio, number of nuclei, nucleus shape and localization as well as chromatin staining and cytoplasm characteristics, including dark to light staining, the presence of granules, the visualization of Golgi, etc.

### 2.5. Fluorescent-Antibody Staining of Bone Marrow Cells

The PFA-fixed bone marrow cells on cytospin slides were soaked in PBS for 5 min, transferred to 100% methanol (−20 °C) for 3 min, and blocked in 5% BSA for 60 min. Next, the slides were washed in PBS and stained with DyLight 488-conjugated rat monoclonal anti-mouse CD34 and with allophycocyanin-conjugated rat monoclonal anti-mouse CD117 (1:250 and 1:100 in 1% BSA, respectively; both from Novus Biologicals, Centennial, CO, USA), as previously described [26,27,28,29,30,31,32]. The slides were kept in a humidity chamber for 90 min at room temperature. After washing in PBS for 5 min, a coverslip with a glycerol-mounting medium was mounted on a dry microscope slide, and the cells were imaged and analyzed under a confocal microscope (NikonD-Eclipse C1; Nikon, Japan) with a 60× objective. The ability of the confocal microscope to block out-of-focus light and thereby perform optical sectioning through a specimen allowed for the fluorescence to be quantified with very high precision [34]. The relative fluorescence of CD34 and CD117 on the cells was assessed as the mean fluorescence intensity (MFI) using ImageJ software, which is a reliable method for repeatedly quantifying hundreds of images and is the method of choice when there is a limited number of tissue samples [27]. All experiments were performed in triplicate.

### 2.6. Cytokine Measurement

Mice were euthanized as indicated earlier, and blood was collected via heart puncture. Serum was prepared and stored at −70 °C. Using the multiplex bead immunoassay (Bio-Plex Pro™ Mouse Cytokine Th1/Th2 Panel, 8-plex, Bio-Rad, Hercules, CA, USA), the following cytokines were examined simultaneously in the serum samples, according to the manufacturer’s protocol: interleukin (IL)-2, IL-4 IL-5, IL-10, IL-12, granulocyte macrophage-colony stimulating factor (GM-CSF), TNF-α, and IFN-γ. Data were acquired using the Bio-Plex^®^ MAGPIX™ Multiplex Reader (Bio-Rad, Hercules, CA, USA) and analyzed using Bio-Plex Data Pro™ Software (Bio-Rad, Hercules, CA, USA). The sensitivity of the test for individual biomolecules was 0.6 pg/mL for IL-2, 0.3 pg/mL for IL-5, 1 pg/mL for IL-10, 2.3 pg/mL for IL-12(p70), 5.6 pg/mL for GM-CSF, 1.4 pg/mL for TNF-α, and 1.2 pg/mL for IFN-γ.

### 2.7. Isolation of Alveolar Macrophages and Assessment of Phagocytosis

Alveolar macrophages were isolated by the infusion of Ca^2+^- and Mg^2+^-free PBS (Biowest, Nuaillé, France) containing 0.5 mM EDTA into the lung through the trachea using a 20 G catheter (Kruuse, China) attached to a syringe. The collected bronchoalveolar lavage (BAL) fluid (∼3 mL) was centrifuged at 1200 rpm/4 °C for 10 min, and the pellet was resuspended in complete RPMI-1640 medium (Sigma-Aldrich, Steinheim, Germany) supplemented with 10% fetal bovine serum (FBS) (Biowest, France), 2 mM L-glutamine (Sigma-Aldrich), 100 U/mL penicillin (Sigma-Aldrich), and 100 μg/mL streptomycin (Sigma-Aldrich). Macrophages (2 × 10^5^) were plated on a cover slip and incubated overnight at 37 °C/5% CO_2_. Afterwards, to remove any undetached cells, the cover slip was rinsed twice with PBS, and 1 × 10^7^ live *M. bovis* BCG bacilli (Biomed, Lublin, Poland), at the multiplicity of infection (MOI) of 50:1 was added to complete RPMI-1640 medium. Three hours later, the medium was removed, and the cover slip was gently rinsed with PBS. The cells were fixed for 10 min with 80% methanol, stained for 15 min with 0.01% acridine orange (Sigma-Aldrich, Steinheim, Germany), and quenched with 0.002% methylene blue (POCH, Gliwice, Poland). Finally, the coverslips were mounted onto glass slides, and the cells were analyzed with fluorescence microscopy to determine bacterium engulfment (Axio Scope.A1, Carl Zeiss, Germany).

### 2.8. Staining of Splenocytes and Flow Cytometry for the Assessment of Central Memory T Cells (T_CM_) and Effector Memory T Cells (T_EM_)

Spleens were isolated from the euthanized animals, placed into ice-cold culture medium in Petri dishes, and stored on ice. Each spleen was homogenized, transferred to a 15 mL conical tube, and centrifuged at 350× *g* for 10 min (+4 °C). The pellet was resuspended in 1 mL of red blood cells lysis buffer (BioLegend, San Diego, CA, USA) for 5 min. A 10 mL amount of RPMI-1640 medium was added to stop the reaction. After centrifugation, the pellet was resuspended in 1 ml of PBS, and the cells were counted and stained as previously described [23]. Briefly, the splenocytes were fixed to assess CD4 and CD8 with the following mAb: APC-Cy7-anti-CD4 and APC-anti-CD8 (BD Biosciences, San Diego, CA, USA), and to determine the memory T cell markers: APC-Cy7-anti CD4, APC-anti-CD8, V500-anti-CD44 (BD Biosciences, San Diego, CA, USA), PE-Vio770-anti-CD62L (Miltenyi Biotec, Bergisch Gladbach, Germany), and FITC-anti-CD127 (eBiosciences, San Diego, CA, USA). An irrelevant isotype-matched mAb was used as the control. Stained cells were incubated for 30 min at 4 °C. To differentiate between splenocytes with the T_CM_ and T_EM_ phenotype, we used a previously published gating strategy based on the expression of the CD62L, CD44, and CD127 markers (Figures S1–S3) [23].

The central memory T cells (T_CM_) were identified as CD62L^+^CD44^+/−^ CD127^+^ CD4/CD8^+^, and the effector memory T cells (T_EM_) were identified as CD62L ^−^ CD44^+^CD127^+/^^−^ CD4/CD8^+^. Data were acquired and analyzed using a BD^®^ LSR II Flow Cytometer (BD Biosciences, San Jose, CA, USA) and FlowJo software. A minimum of 10,000 events were collected. The results are presented as a percentage of positive spleen cells (% gated). 

### 2.9. Statistical Analysis

The statistical significance of the differences was determined by using Statistica 13.1 PL software (Statsoft). After verifying the assumptions (including normality using the Kolmogorov–Smirnov test and homogeneity of variance with the Levene test as well as the type of data and the number of data), parametric and non-parametric tests were used. The one-way analysis of variance (ANOVA) with Tukey’s post hoc test and Kruskal–Wallis test, respectively, were used to determine the differences between antigens. *p* values < 0.05 were considered significant.

## 3. Results

### 3.1. Composition of Leukocytes in Cytospins from Bone Marrow Cells

**Figure 1:**
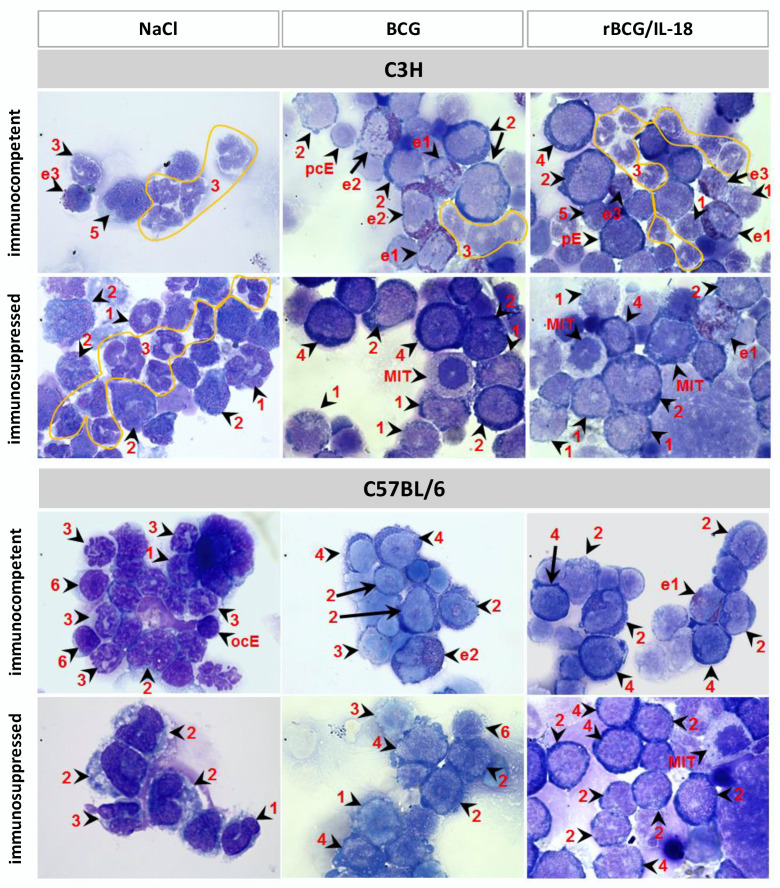
Morphology of bone marrow cells isolated from C3H and C57BL/6 mice who had been immunocompromised with cyclophosphamide or immunocompetent animals who had been injected with physiological saline (NaCl) and immunized with *Mycobacterium bovis* (wild-type BCG) or the recombinant BCG (rBCG) strain secreting interleukin (IL)-18. Representative pictures are shown for each group. Cytospins were prepared from freshly isolated bone marrow and stained with the May–Grünwald for 6 min and Giemsa solution for another 15 min. Finally, the slides were rinsed with water and placed to dry in air. The cells were observed using a light microscope at 100× magnification (immersion). The experiment was triplicated, and each time, at least 12 randomly selected view fields per slide were captured. The cells were identified on the basis of histological/morphological markers by two independent histopathologists from Institut für Tierpathologie, Berlin, Germany, and are indicated by arrows and numbers: (1) myelocyte, (2) promyelocyte, (3) metamyelocyte, (4) myeloblast, (5) monocyte, (6) lymphocyte, (e1) eosinophilic myelocyte, (e2) eosinophilic promyelocyte, (e3) eosinophilic metamyelocyte, (MIT) mitosis, (pE) proerythroblast, (pcE) polychromatic erythroblast, and (ocE) orthochromatic erythroblast.

The cytospin slides of the bone marrow from all of the experimental variants developed in mice (mice non-immunized or immunized with the wild-type BCG or rBCG/IL-18 and those that were immunocompetent or immunocompromised with CTX) were evaluated for the percentage (%) of granulocytes, macrophages, and lymphocytes as well as to determine the different granulocyte developmental stages: myeloblasts, promyelocytes, myelocytes, and metamyelocytes. (Figure 1). There were no statistical differences in the total numbers of immune cells (granulocytes, monocytes, and lymphocytes) in mice who had been immunized with the wild-type BCG or rBCG/IL-18 or those who were non-immunized, immunocompetent, or immunocompromised (Table 1). However, in the C57BL/6 mice who were non-immunized or who had been immunized with mycobacteria, the number (%) of lymphocytes was diminished after CTX administration (immunocompetent mice: 5.7; 3.2; 3.5, respectively vs. immunocompromised mice: 0.0; 1.6; 0.0, respectively).

We then analyzed how the vaccination of immunocompetent C57BL/6 (Bcgs) and C3H (Bcgr) mice with the wild-type BCG or rBCG/IL-18 influenced the number of myeloblasts being the precursors of granulocytes and monocytes as well as the number of granulocyte maturation stages: promyelocytes, myelocytes, and metamyelocytes. The same analysis was carried out in mice who had been injected with CTX only or who had been vaccinated with mycobacteria and then treated with an immunosuppressant. The percentage of myeloblasts, promyelocytes, and metamyelocytes differed significantly depending on the mouse strain and the mode of bacteria and/or CTX vaccination (Table 1). 

In the C57BL/6 (Bcgs) mice who were non-immunized or inoculated with the wild-type BCG or rBCG/IL-18, the metamyelocyte percentages (premature granulocytes) were 38.7%, 39.7%, and 45.7%, respectively (Table 1). The number of such cells after CTX administration increased to 59% in the non-immunized mice, suggesting their propagation in response to the immunosuppressant. In the C57BL/6 mice who had been vaccinated with the wild-type BCG or rBCG/IL-18 and who later received CTX administration, the levels of premature granulocytes were only 13% and 10%, respectively, with 43.8% and 42.4% of the promyelocytes at the same stage, respectively. In immunosuppressed C57BL/6 mice who had been inoculated with rBCG/IL-18, the number of myeloblasts (18.6%) was also increased compared to in the mice who had been treated with CTX alone (5.7%). In the C3H (Bcgr) mice who were non-immunized with mycobacteria, the number of metamyelocytes was higher (61.6%) than it was in non-immunized C57BL/6 mice (38.7%). After the immunization of the C3H mice with the wild-type BCG or rBCG/IL-18, the metamyelocyte levels decreased from 61.0% in the control (NaCl) mice to 41.5% and to 44.9% in the immunized individuals, respectively, similar to what was observed in the non-immunized CTX-injected mice (43.0%). However, in the C3H mice who had been immunized with the wild-type BCG or rBCG/IL-18 followed by CTX administration, the number of metamyelocytes decreased to 4% and 10%, respectively, whereas the promyelocyte levels reached 43.9% and 44.9%, respectively. In both of the mice strains that were inoculated with the wild-type BCG or rBCG/IL-18 and treated with CTX, the number of myeloblasts tended to grow, and this growth was mainly driven by mycobacteria. These results may indicate the initiation of an inflammatory response or the interference of mycobacteria during the maturation of myeloblast-derived cell lines (granulocytes and monocytes). Promyelocyte propagation was mainly triggered by CTX in the mice who had been vaccinated with the wild-type BCG or BCG/IL-18 and then immunosuppressed, which means that both the CTX and mycobacteria were responsible for such an effect.

### 3.2. CD34 and CD117 Expression on Bone Marrow Cells

**Figure 2:**
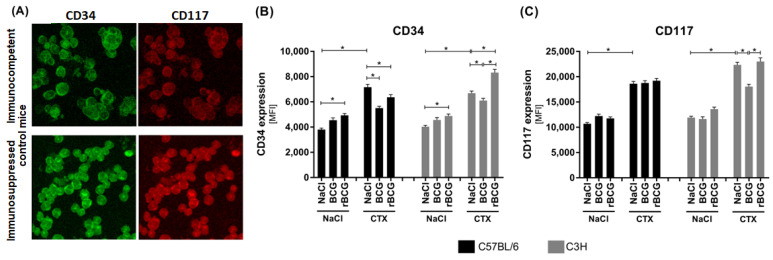
The expression of CD34 and CD117 on bone marrow cells in C57BL6 and C3H mice, in immunocompetent mice and in mice who were immunocompromised with cyclophosphamide (CTX), and in mice who were non-immunized or immunized with *Mycobacterium bovis* wild-type BCG or by the recombinant BCG (rBCG) strain secreting IL-18. (**A**) Representative confocal laser scanning microscopy images (NikonD-Eclipse C1 microscope equipped with an inverted 60× objective). Bone marrow cells were stained with DyLight 488-conjugated rat monoclonal anti-mouse CD34 (**B**) and with allophycocyanin (APC)-conjugated rat monoclonal anti-mouse CD117 (**C**). Data are shown as mean ± SEM (standard error of the mean). Asterisk (*) indicates *p* value ≤ 0.05; NaCl—physiological saline solution NaCl 0.9%; CD—cluster of differentiation; MFI—mean fluorescence intensity.

On bone marrow cells, there are two surface functional molecules that are typical of hematopoietic stem cells, CD34 and CD117, and these were determined in the C57BL6 and C3H mice, in those immunocompetent and immunocompromised with CTX, and in the mice who were non-immunized or immunized with *Mycobacterium bovis* wild-type BCG or rBCG/IL-18 (Figure 2).

Both mice of the strains that had been vaccinated with *M. bovis* bacilli (wild-type BCG or rBCG/IL-18) vs. the non-immunized mice (NaCl) showed an increased CD34 expression on the bone marrow cells (Figure 2A,B). The difference in the rBCG/IL-18-vaccinated animals was statistically significant, whereas an upward trend could be observed in the animals that had been inoculated with the wild-type BCG (Figure 2B). In both the C57BL/6 and C3H mice who were non-immunized with mycobacteria but treated with CTX alone, the CD34 expression on the bone marrow cells was higher than it was in the control immunocompetent mice (NaCl), which may indicate that CTX was an independent causative agent (Figure 2A,B). CD34 expression was also increased on the bone marrow cells from the C57BL/6 or C3H mice who had been inoculated with either the wild-type BCG or rBCG/IL-18 and then injected with CTX compared to the cells from the immunocompetent animals who had been immunized with mycobacteria and not immunocompromised. However, in the C57BL/6 mice who had been vaccinated with mycobacteria, the CD34 expression was reduced compared to that in the animals who had only been injected with CTX, although the expression remained higher than it did in the immunocompetent animals who had been injected with the wild-type BCG or rBCG/IL-18 (Figure 2B). This means that the vaccine bacilli and CTX upregulated the CD34 expression independently. In the C3H mice who had been immunized with rBCG/IL-18 and who were immunosuppressed, the CD34 deposition was even higher than it was in mice receiving CTX only, indicating that rBCG/IL-18 was a strong stimulator of CD34 expression in this group of mice (Figure 2B). 

In the immunocompetent mice from both strains, the CD117 expression on the bone marrow cells did not change after vaccination with mycobacteria (Figure 2C). In mice who had only been treated with CTX or who had been inoculated with mycobacteria and then injected with CTX, the CD117 expression was significantly higher than it was in the immunocompetent animals. Only in the C3H mice, which were vaccinated with the wild-type BCG and then treated with CTX, was the level of CD117 lower than it was in the non-immunized but immunosuppressed mice (Figure 2C). 

These results reveal that in C57BL/6 and C3H immunocompetent mice, vaccination with rBCG/IL-18 increased CD34 expression on the bone marrow cells. CTX alone also induced CD34 expression on such cells. This indicates that mycobacteria and CTX can drive CD34 expression independently. In the C3H mice who had been inoculated with rBCG/IL-18 and then exposed to the immunosuppressant, we could see a synergistic effect of both. In the case of CD117, this molecule was upregulated by CTX rather than by mycobacteria.

### 3.3. Phagocytic Activity of Alveolar Macrophages

**Figure 3:**
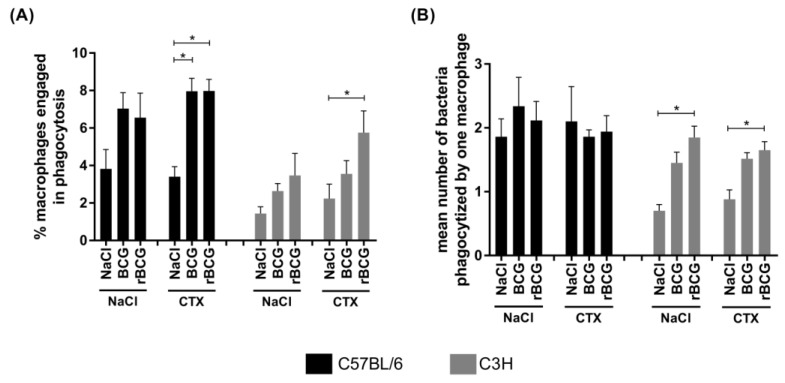
Phagocytic activity of alveolar macrophages in C57BL6 or C3H mice who were immunocompetent or immunocompromised with cyclophosphamide (CTX) and who were non-immunized or immunized with *Mycobacterium bovis* wild-type BCG or the recombinant BCG strain (rBCG) secreting IL-18. The percentage of macrophages with at least one engulfed bacterium (**A**). The mean number of bacteria that were phagocytized by one macrophage (**B**). At least three hundred of alveolar macrophages were counted in each slide. The number of ingested bacteria and the number of phagocytes containing at least one bacterium were determined. The percentage of macrophages engaged in phagocytosis (with at least 1 bacterium) was calculated as follows: (number of phagocytes containing bacteria/total number of phagocytes counted in slide) × 100%. The mean number of bacteria phagocytized by one macrophage was calculated as follows: total number of ingested bacteria/number of phagocytes containing at least one bacterium. Data are shown as mean ± SEM (standard error of the mean). Differences were calculated using a Kruskal–Wallis test, and statistical significances are indicated vs. control mice. Asterisk (*) indicates *p* value < 5; NaCl—physiological saline solution NaCl 0.9%.

The phagocytic activity of the alveolar macrophages from the immunocompetent or immunosuppressed mice who had been immunized with the wild-type BCG or with rBCG/IL-18 stimulated with live *M. bovis* BCG bacilli *in vitro* was assessed using the acridine orange staining method. In general, the number of phagocytes capable of engulfing mycobacteria was higher in the C57BL/6 mice than it was in the C3H mice (Figure 3A). In both mouse strains, regardless of whether the mice were immunized with the wild-type BCG or rBCG/IL-18, the number of phagocyte-ingesting mycobacteria was increased compared to in the control (non-immunized mice); however, the difference was not statistically significant (Figure 3A). 

The CTX alone did not increase the number of cells capable of mycobacteria phagocytosis compared to the non-immunized control mice from both strains; however, the number of phagocytizing macrophages was significantly increased in the mice who had been vaccinated with the wild-type BCG or rBCG/IL-18 and who had been treated with CTX. This indicates that phagocytosis was driven in response to infectious agents (Figure 3A); however, in the C3H mice who had been injected with rBCG/IL-18 and then received CTX, the number of phagocytizing macrophages was higher than it was in the immunocompetent control mice (NaCl), (Figure 3A). These results indicate that the alveolar macrophages from immunocompetent as well as from CTX-immunocompromised mice were able to engage in phagocytosis in response to immunization with mycobacteria. It is interesting that the number of phagocytizing macrophages in the C3H mice was found to increase, especially in the mice who had been inoculated with rBCG/IL-18 and treated with CTX (Figure 3A), which was corelated with the ability of the macrophages to engulf more bacteria (Figure 3B). In the C57BL/6 mice, the number of macrophages involved in phagocytosis increased significantly after inoculation with mycobacteria and CTX administration (Figure 3A); however, the effectiveness of phagocytosis, which is measured as the number of ingested bacteria, was similar after the animals had been treated with the different BCG formulations and CTX (Figure 3B). This might be due to the macrophages of the immunocompetent C57BL/6 mice having a higher initial phagocytic activity (NaCl 0.9%), which might be a limitation in our experimental model.

### 3.4. Serum Cytokine Concentration

**Figure 4:**
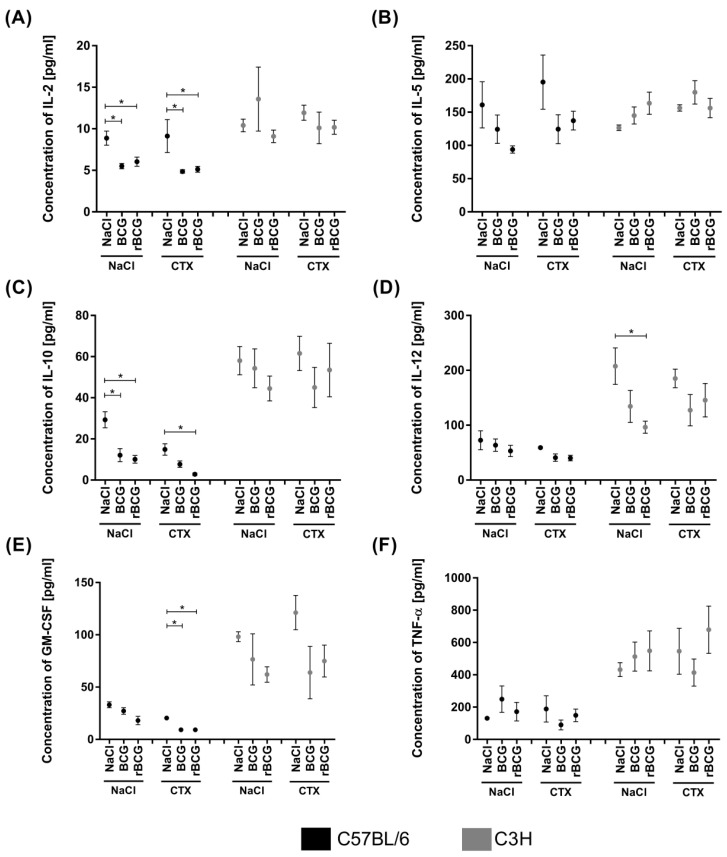
Serum concentrations of the studied cytokines in the sera from C57BL/6 and C3H mice who had been immunized with wild-type BCG or the recombinant BCG strain (rBCG) secreting IL-18 and who were immunocompetent or cyclophosphamide (CTX)-compromised. Data are shown as mean ± SEM (standard error of the mean). For each condition, samples were tested. *p* values were calculated using a one-way analysis of variance (ANOVA). IL-2 (**A**), IL-5 (**B**), IL-10 (**C**), IL-12 (**D**), granulocyte macrophage colony stimulating factor (GM-CSF) (**E**), tumor necrosis factor alpha (TNF)-α (**F**). Black dots—C57BL/6 mice; grey dots—C3H mice. Asterisk (*) indicates *p* value ≤ 0.05.

The cytokine levels, including those of the Th1 cytokines: IL-2, IL-12, TNF-α, and IFN-γ, and the Th2 cytokines: IL-4, IL-5, and IL-10, as well as GM-CSF were determined in the sera from immunocompetent and immunocompromised C3H and C57BL/6 mice who had been immunized with the wild-type BCG or rBCG/IL-18. In both mouse strains, the IFN-γ and IL-4 levels were undetectable (below the assay’s sensitivity). In general, the levels of other cytokines were higher in the C3H (Bcgr) mice than they were in the C57BL/6 (Bcgs) mice (Figure 4). In both mice strains, TNF-α production was not significantly affected in the immunocompetent or immunosuppressed mice, regardless of whether they were non-immunized or immunized with mycobacteria (Figure 4F). 

In the C57BL/6 mice who had been inoculated with mycobacteria, the IL-2 and IL-10 levels were significantly lower than they were in the non-immunized mice (Figure 4A,C). The IL-5, IL-12, and GM-CSF levels were also lower in the animals who had been inoculated with mycobacteria than they were in the control mice; however, the differences were not statistically significant (Figure 4B,D,E). CTX administration did not diminish the spontaneous production of these cytokines; however, the IL-2, IL-10, and GM-CSF levels were significantly reduced in the mice who had been inoculated with mycobacteria and then with CTX (Figure 4A,C,E). 

In the C3H (Bcgr) immunocompetent mice, only the IL-12 level diminished significantly after inoculation with rBCG/IL-18 (Figure 4D). CTX administration did not significantly influence cytokine production in the non-immunized C3H mice or in the mice who had been vaccinated with mycobacteria (Figure 4A–F). 

### 3.5. Effect of BCG and rBCG on the CD4^+^ and CD8^+^ T_CM_ and T_EM_ Lymphocytes Response

**Figure 5:**
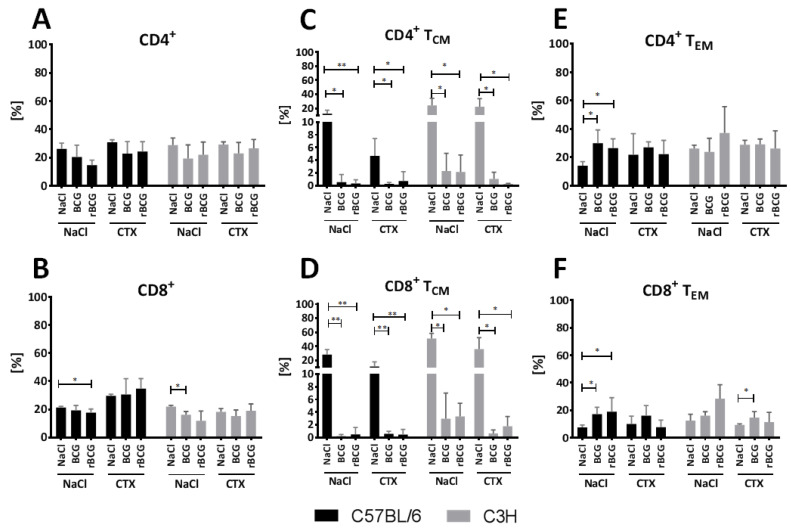
Comparison of central (T_CM_) and effector memory T cells (T_EM_) in C57BL6 and C3H mice who were non-immunized or immunized with the wild-type BCG or recombinant BCG (rBCG) secreting IL-18 and in mice who were immunocompromised with cyclophosphamide (CTX) or who were immunocompetent (NaCl, Control). Graphs show cumulative data for *n* = 7 mice/group. Data are expressed as mean ± SD (standard deviation) T_CM_ or T_EM_ percentages; * *p* value < 0.05, ** *p* value < 0.01. (**A**) total number of CD4^+^ T cells, (**B**) total number of CD8^+^ T cells, (**C**) number of CD4^+^ T_CM_ cells, (**D**) number of CD8^+^ T_CM_ cells, (**E**) number of CD4^+^ T_EM_ cells, (**F**) number of CD8^+^ T_EM_ cells.

To determine the effects of BCG or rBCG/IL-18 vaccination on the population of T lymphocytes belonging to the CD4^+^ or CD8^+^ phenotypes and the population of central memory T cells (T_CM_) or effector memory T cells (T_EM_) from both phenotypes in mice who were sensitive (C57BL/6) or resistant (C3H) to BCG, the splenocytes from immunocompetent and CTX-immunocompromised mice who were non-immunized or immunized with the wild-type BCG or rBCG/IL-18 were analyzed by flow cytometry after staining the specific CD molecules. The flow results are shown in Figure 5. 

The total number of CD4^+^ T cells from both strains of mice treated with the wild-type BCG or with rBCG/IL-18 was similar to the total number of CD4-positive cells in the control mice (NaCl). CTX alone did not influence CD4 expression in either mouse strain. In the mice who had been vaccinated with mycobacteria and then treated with CTX, the CD4 expression that was induced by the mycobacteria was not affected as a result of CTX administration. 

In the immunocompetent mice, the number of CD4^+^ T_CM_ splenocytes was significantly decreased after vaccination with the wild-type BCG or rBCG/IL-18 (Figure 5C). The number was also not diminished after CTX administration; however, in the mice who had been vaccinated with mycobacteria and then treated with CTX, the number of CD4-positive T_CM_ cells was significantly lower than it was in the mice who had been injected with CTX only (Figure 5C). This might indicate that mycobacteria were the causative agent, diminishing the number of CD4^+^ T_CM_ splenocytes. 

When analyzing the CD4^+^ T_EM_ splenocytes in the C57BL/6 but not in the C3H mice, we could see a significantly increased number of positive cells after vaccination with the wild-type BCG or rBCG/IL-18 (Figure 5E). CTX administration did not influence the frequency of positive cells in both mouse strains. The increased number of CD4^+^ T_CM_ cells in the C57BL/6 mice in response to vaccination with mycobacteria was restored in the mice who received CTX (Figure 5E).

As indicated in Figure 5B, the total number of CD8^+^ cells was significantly lower in the C57BL/6 mice who had been injected with rBCG/IL-18 and in the C3H mice who had been injected with the wild-type BCG compared to the control mice (NaCl). In the C57BL/6 strain but not in the C3H strain, CTX administration increased the number of CD8-positive splenocytes vs. immunocompetent mice; however, the difference was not statistically significant (Figure 5B). Similarly, in the C57BL/6 mice who had been vaccinated with mycobacterial formulations followed by the administration of CTX, the number of CD8 positive cells was found to be increased (Figure 5B).

In the case of both immunocompetent mouse strains, the number of CD8^+^ T_CM_-positive splenocytes was significantly decreased in the animals who had been vaccinated with the wild-type BCG or rBCG/IL-18 (Figure 5D). CTX alone did not reduce the number of CD8^+^ T_CM_ cells; however, in the mice who had been vaccinated with mycobacteria and treated with CTX, the number of CD8^+^ T_CM_ cells was considerably reduced, which might have been related to vaccination with the different BCG formulations (Figure 5D).

As far as the number of CD8^+^ T_EM_ splenocytes is concerned, in the C57BL/6 mice, we could see an increased number of positive cells after vaccination with the wild-type BCG or with rBCG/IL-18 (Figure 5F). In the C3H mice, only an increasing trend was observed. In both mouse stains, CTX alone did not influence the amount of CD8^+^ T_EM_. In the mice who were vaccinated with mycobacteria after the administration of CTX, we could see a weak decrease in the levels of positive cells; however, the difference was not statistically significant (Figure 5F). 

## 4. Discussion

The aim of this study was to establish the immune status of mice who were susceptible (C57BL/6, Bcgs) or resistant (C3H, Bcgr) to BCG mycobacteria in response to immunization with the wild-type BCG or rBCG/IL-18 secreting mouse IL-18, which is a prototype of a new BCG formulation. To assess whether the wild-type BCG or rBCG/IL-18-driven immune responses could be developed and persist when immune mechanisms are weakened, we used our previously developed model of immunocompetent or CTX immunosuppressed mice [23]. Our previous study (data not published) revealed that the immunization of mice with the wild-type BCG resulted in an enlargement of the cortical zone accompanied by an increase in the number of lymphocytes and plasma cells in the medulla of draining lymph nodes. This could be due to the migration of lymphocytes to the antigen presentation sites within the lymph nodes [35]. On the contrary, the mice who were inoculated with the wild-type mycobacteria and then with CTX showed a reduction in the cortical zone and an increase in the number of macrophages in the medulla as well as pigmented macrophages and granulocytes in the paracortical area. Histological examinations of the lymph nodes showed a reduction in the number of follicles in the cortex in the mice who had been inoculated with mycobacteria and CTX. The functional consequence of this was a complete inhibition of the production of antibodies against BCG bacilli. These two experimental mice models, C57BL/6, Bcgs and C3H, Bcgr, may help to demonstrate differences in the immune processes driven by the immunization of animals with the wild-type BCG or rBCG/IL-18 in immunocompetent mice and to assess the influence of CTX administration on the immune status induced by vaccine mycobacteria. The assessed parameters included the total number of immune cells in the bone marrow: granulocytes, macrophages, and lymphocytes, as well as the number of myeloblasts (precursors of granulocytes and monocytes), and the developmental stages of the granulocytes that initiate the inflammatory response. 

There were no statistical differences in the total numbers of immune cells in the bone marrow in the mice who had been immunized with the wild-type BCG or rBCG/IL-18 or in the non-immunized mice, regardless of whether they were immunocompetent or immunocompromised (Table 1). Similarly, Cirovic et al. [36] did not find any differences within the total number of leukocytes in the peripheral blood and the bone marrow in individuals who had been vaccinated with BCG or those who were non-vaccinated [36]. In immunocompetent C57BL/6 mice, regardless of whether they had been non-immunized or immunized with mycobacteria, the lower number of bone marrow lymphocytes after CTX administration might be due to a different sensitivity to CTX between mice strains. No differences were observed between the immunocompetent and immunocompromised mice in terms of the number of immunocompetent cells in the current study, and this could depend on the CTX dose [23,37,38]. It is also possible that bone marrow cells and peripheral blood leukocytes may differ in terms of their sensitivity to CTX [23]. 

No differences were observed regarding the percentage of immune cells upon wild-type BCG or rBCG/IL-18 vaccination in immunocompetent or immunocompromised mice, and this prompted us to analyze bone marrow cell subpopulations: myeloblasts, promyelocytes, myelocytes, and metamyelocytes, in terms of their maturity. In mice from both strains who were vaccinated with the wild-type BCG or rBCG/IL-18, there was an increased number of metamyelocytes (pro-mature granulocytes) and myeloblasts. In the mice receiving mycobacteria and then CTX or CTX alone (only in C3H mice), promyelocytes were dominant. Buisman et al. [39] showed that CTX predominantly affects the later cell division stages in the bone marrow, resulting in a decrease in the number of granulocytic cells, monocytic cells, lymphoid cells, and myeloid blasts [39]. The differences in the number of maturation stages in the bone marrow cells between various studies might be due to different examination methods.

Hematopoietic cells respond to specific growth factors by means of proliferation and differentiation. In our study, the serum concentration of GM-CSF was diminished in the mice who had been vaccinated with mycobacteria and further decreased in the animals who received CTX. It is possible that bone marrow cells, which were not affected by the CTX resting phase, were triggered to proliferate faster [39]. In the mice who had been inoculated with mycobacteria, the production of monoblasts and of granulocyte precursors in particular, was induced, and these precursors were then propagated by CTX. Using a C57BL/6 mice model, Buisman et al. [39] showed that CTX spared G-CSF responsive cells, thus enabling the enhanced G-CSF-mediated cell recovery [39]. Whether the propagation of monoblasts and myeloblasts that we observed in mice in response to mycobacterial vaccination and CTX treatment was related to GM-CSF induction was not confirmed. The amount of GM-CSF was decreased in the serum samples in the mice who were vaccinated with mycobacteria, who were immunocompetent, or who were treated with CTX. An accumulation of granulocyte precursors in the bone marrow may result in a systemic release of immature cells. Roberts and Metcalf [40] showed an increase in the number of immature circulating granulocytes after CTX treatment, regardless of whether it was combined with GM-CSF [40]. 

We examined the hematopoietic stem cell markers CD34 and CD117 on bone marrow cells [26,27,28,41,42]. CD34 enhances the proliferation of hematopoietic cells and blocks differentiation and CD34 is progressively lost as hematopoietic cells mature [26]. Fackler et al. [32] showed that the expression of CD34 in the myelomonocytic cells was able to block terminal differentiation into macrophages and to maintain the cells in a highly proliferative state [32]. The CD34 expression in the C57BL/6 and C3H mice increased significantly in the mice who were vaccinated with rBCG/IL-18. CTX alone also upregulated CD34 expression; however, when using C57BL/6 mice, Feng et al. [29] reported the opposite effect [29]. In our study, in the C3H mice who had been inoculated with rBCG/IL-18 and treated with CTX, a further increase in CD34 expression was observed, which may indicate that IL-18 might be responsible for this effect. IL-18 stimulates hematopoietic growth factors and augments the number of circulating granulocytes in mice [30]. It would be interesting to determine whether there was a difference in the maturity of the circulating granulocytes in the mice who had been vaccinated with the wild-type BCG or rBCG/IL-18 and then treated with CTX. Recently a type of CD15^+^ granulocytes termed low-density granulocytes (LDGs) was found in the peripheral blood in humans and were found to potentially play an unfavorable role in the pathogenesis of tuberculosis [31]. Mycobacterial vaccines did not increase CD117 expression in bone marrow cells by themselves; however, we could see significantly increased CD117 deposition and maintenance in the mice who had been treated with CTX alone or in immunosuppressed mice who had been vaccinated with mycobacteria. These results were compatible with the data on the propagation of early developmental stages of myelocyte lineage. Hematopoietic cell interactions, particularly those with stromal cells and mediated by integrins, may deliver additional signals for cell expansion and/or differentiation. 

CTX mainly affects T lymphocytes, which secrete cytokines that activate macrophages. The lack of or a lower amount of T cell-derived cytokines may result in macrophages being less effective in pathogen elimination [38,43]. We assessed the number of alveolar macrophage-ingesting mycobacteria ex vivo and the effectiveness of engulfment (number of ingested bacteria per single macrophage) in mice who were either non-vaccinated or vaccinated with the wild-type BCG or rBCG/IL-18, who were immunocompetent, or who were CTX-treated. The number of macrophages involved in phagocytosis was increased to the same level in both the C57BL/6 and C3H mice who had been immunized either with the wild-type BCG or rBCG/IL-18 compared to mice receiving NaCl. CTX alone did not affect phagocytosis. Lis et al. [44] showed that the single i.p. injection of CTX at a dose of 350 mg/kg did not change the percentage of the phagocytosing monocytes; however, CTX inhibited the production of reactive oxygen metabolites [44]. In our study CTX did not influence the number of macrophages engaged in phagocytosis in the C57BL/6 mice who were inoculated with mycobacteria, while in the C3H mice who had been vaccinated with rBCG/IL-18 and then received CTX, the number of phagocytosing macrophages increased further. This might have been due to the development of a specific immune milieu in the lungs in C3H (Bcgr) mice who had been vaccinated with rBCG/IL-18 mycobacteria producing IL-18. The C57BL/6 (Bcgs) macrophages ingested more mycobacteria than the C3H (Bcgr) macrophages of the mice did. The effectiveness of bacterial engulfment was not affected in the C57BL/6 mice due to vaccination with mycobacteria and/or CTX administration. In the C3H mice, both the number of phagocytes engaged in phagocytosis and the effectiveness of engulfment increased significantly after inoculation with rBCG/IL-18. This effect was maintained in the immunocompromised mice. These differences in phagocytic potential of the macrophages of the C57BL/6 vs. the C3H mice towards mycobacteria might represent the genetic background. The lack of difference in the number of bacteria engulfed by one macrophage between immunocompetent and immunocompromised C56BL/6 mice might be the result of macrophage sensitivity to CTX. Potentially, the higher natural phagocytic activity observed in the macrophages of the C57BL/6 mice than the C3H mice might be a limitation in this study. The difference in phagocytosis induction between the wild-type BCG and rBCG/IL-18 could be related to IL-18. It was shown that IL-18 enhances the production of IFN-γ by the immune cells, which may result in the macrophages having increased antibacterial activity [45,46]. Unfortunately, IFN-γ was not detected in the serum of any of the experimental mice variants, which might be due to the low sensitivity of the assay or the temporary immunosuppression driven by mycobacteria. In the study by Biet et al. [9], higher amounts of IFN- γ were detected in the cell cultures of splenocytes or lymph node lymphocytes from mice who had been vaccinated with rBCG/IL-18 than with the wild-type BCG, but not in the serum [9]. Thus, our results remain in accordance with data of Biet et al. [9]. Steenwinkel et al. [47] showed a significant decline in the systemic level of IFN-γ between four and nine weeks after the mice had been inoculated with M.tb [47]. In M.tb patients, a diminished level of IFN-γ is corelated with an increased number of activated Foxp3^+^ regulatory T (Treg) lymphocytes and the production of transforming growth factor (TGF)-β [48,49]. It has been shown that IL-18 induces TGF-β production [50], which might stimulate natural killer cells to deliver tristetrapolin, which is responsible for IFN-γ degradation [51]. It was also revealed that endogenous IL-12 plays a favorable role in rBCG/IL-18-induced IFN-γ production [52]. In the current study, the systemic level of IL-12 was significantly diminished, particularly in C3H mice who had been vaccinated with rBCG/IL-18. Schultz et al. [53] showed that IL-18 impaired the pulmonary host response to *Pseudomonas aeruginosa*, whereas Ghose et al. [54] revealed that the interaction between the IL-18 and IL-18 receptors limited the protective immunity towards *Ehrlichia* [53,54]. 

The immune responses to infectious agents are regulated by various cytokines [55,56]. We compared the concentration of the proinflammatory cytokines (TNF-α, IL-2, IL-12, IFN-γ) and anti-inflammatory cytokines (IL-4, IL-5, IL-10) delivered by T helper 1 (Th1) or Th2 lymphocytes, respectively, as well as the GM-CSF level. The IFN-γ and IL-4 levels were undetectable, which might be due to the low sensitivity of the assay or the negative regulation of these cytokines in our model. The C3H mice (Bcgr) were more effective cytokine producers than the C57BL/6 mice (Bcgs) were. Literature data show that inbred mouse strains respond to mycobacterial infection via the distinct polarization of T helper-dependent processes and that the immune responses of C57BL/6 and C3H mice are Th1 prone [57,58]. It is not clear whether vaccinating mice with rBCG/IL-18 is beneficial, especially when taking into account the significant downregulation of the cytokine response in this study. This could potentially be due to secreted IL-18. Further studies are needed to elucidate the role of rBCG/IL-18 in regulating cytokine production in response to mycobacteria. The diminished production of cytokines in immunized mice treated with CTX indicate that mycobacteria are able to modify host cytokine responses under the conditions of CTX-induced immunosuppression; however, the influence of immunosuppression is also possible. 

In the study by Kremer et al. [59], the infection of C57BL/6 mice but not of C3H/HeJ mice with BCG induced the massive production of TNF-α in the serum and an increase in Fas and Fas ligand (FasL) expression in the T cells, resulting in a T-cell proliferation defect [59]. In our study, the level of TNF-α was significantly higher in the non-immunized and in the wild-type BCG/rBCG/IL-18-vaccinated mice who were resistant to BCG than it was in susceptible mice, and this did not change due to CTX administration. According to Pelletier et al. [18], resistant mice are able to prevent bacterial growth without the need for a specific cellular response, whereas susceptible mice control bacterial growth by means of cellular immunity [18]. This suggestion might be discussed in the context of IL-2 production, which is a lymphocyte growth factor. The serum level of IL-2 in the C57BL/6 but not in the C3H mice was significantly diminished after vaccination with mycobacteria, and this effect persisted in the mice who received CTX. IL-2 deficiency may limit the development of the cellular adaptive response [50]. 

Only T cells that receive the right amount of stimulation, antigen, and cytokines survive as T_CM,_ which is home to secondary lymphoid organs where they proliferate and differentiate to effector cells, or as T_EM_, which immediately displays an effector function [60,61,62,63]. Additionally, two types of memory CD4 T cells have been identified in mice [64,65]. Our results suggest that in mice who have been vaccinated with wild-type BCG or rBCG/IL-18, the spleen is mainly a reservoir of T_EM_ cells but not T_CM_ cells. Particularly, in the C57BL/6 mice who were injected with mycobacteria, the numbers of CD4^+^ T_EM_ and CD8^+^ T_EM_ cells in the spleen increased and were not diminished after CTX administration. In the BCG-sensitive strain, these cells may express their effector function in response to the challenge. The study by Henao-Tamayo et al. [66] showed that in mice who were vaccinated with BCG, the T_CM_ cells represented a very small population in the spleen, while the CD4^+^ and CD8^+^ T_EM_ cells were mostly generated in the lungs [66]. The presence of T_EM_ lymphocytes in the lungs of mice vaccinated with mycobacteria may drive the effector phagocytic function of macrophages, which become activated by T_EM_-delivered cytokines [66]. The lack or low number of CD4^+^ and CD8^+^ T_CM_ cells in the spleen of mice who have been vaccinated with the wild-type BCG or rBCG/IL-18 may indicate a different homing process for these cells. Further studies are needed to explain whether or not T_CM_ lymphocytes were recruited to the spleen in our model; it is possible that these cells did not propagate due to insufficient signaling, which requires an antigen and cytokines.

## 5. Conclusions

The current study greatly expanded our knowledge of the immune responses to attenuated BCG mycobacteria in immunocompetent mice and in immunocompromised animals, providing a good basis for understanding the potential effect of chemical immunosuppression on the effectiveness of anti-mycobacterial responses in humans. The data that were obtained in the present study confirm the complexity of the immune processes that are involved in the response to mycobacteria. The examined rBCG/IL-18 strain secreting mouse IL-18 increased the number of bone marrow myeloblasts and promyelocytes as well as hematopoietic CD34 adhesin expression in both mouse strains. Furthermore, rBCG/IL-18 mobilized the macrophages for the phagocytosis of mycobacteria, even in the CTX-treated mice, and in the C57BL/6 mice, it induced the spleen homing of effector memory CD4^+^ and CD8^+^ T lymphocytes. In conclusion, rBCG/IL-18 induced selected immune determinants that were maintained in immunocompromised mice.

Further studies on rBCG/IL-18 as a new prototype vaccine strain are needed to evaluate practical implications in the fight against tuberculosis.

## Figures and Tables

**Table 1:** Leukocyte composition (%) of bone morrow cells isolated from C57/BL6 and C3H mice who had been immunized with the wild-type BCG or recombinant BCG secreting IL-18 (rBCG/IL-18) strains and from immunocompetent or cyclophosphamide (CTX) immunosuppressed animals. Data are shown as mean ± SEM (standard error of the mean).

		C57BL/6 Mice						C3H Mice					
		Immunocompetent			Immunosuppressed			Immunocompetent			Immunosuppressed		
		Nacl	BCG	rBCG/IL-18	Nacl	BCG	rBCG/IL-18	Nacl	BCG	rBCG/IL-18	Nacl	BCG	rBCG/IL-18
**Granulocytes**	**Myeloblast**	3.0 ± 0.6	10.4 ± 0.5	5.7 ± 1.7	5.7 ± 1.8	10.2 ± 2.6	18.6 ± 0.6	1.1 ± 1.1	4.8 ± 0.9	5.5 ± 1.4	6.8 ± 1.3	25.5 ± 4.6	18.4 ± 1.5
	**Promyelocyte**	11.4 ± 5.3	12.4 ± 2.7	16.0 ± 4.1	15.7 ± 1.9	43.8 ± 1.4	42.4 ± 0.7	7.7 ± 1.0	18.6 ± 0.7	12.4 ± 2.7	29.2 ± 8.6	43.9 ± 7.5	44.9 ± 3.5
	**Myelocyte**	13.6 ± 1.8	7.7 ± 0.3	11.4 ± 1.8	17.2 ± 2.4	14.1 ± 3.1	19.0 ± 0.3	8.2 ± 0.9	10.9 ± 1.8	12.6 ± 2.6	12.2 ± 4.0	14.1 ± 7.0	19.0 ± 1.7
	**Metamyelocyte**	38.7 ± 5.5	39.6 ± 2.5	45.7 ± 9.9	59.0 ± 2.9	12.9 ± 6.1	10.4 ± 0.7	61.6 ± 4.5	41.5 ± 1.5	44.9 ± 2.9	43.0 ± 15.9	4.0 ± 2.7	10.1 ± 1.6
	**∑**	66.7	70.1	78.8	97.6	81.0	90.4	78.6	75.8	75.4	91.2	87.5	92.4
	**Lymphocytes**	5.7	3.2	3.5	0.0	1.6	0.0	0.0	2.5	2.6	2.0	0.9	0.0
	**Monocytes**	0.9	0.0	1.0	0.0	0.0	0.1	1.3	0.0	0.3	0.0	0.0	0.0
	**∑**	73.3	73.3	83.3	97.6	82.6	90.5	79.9	78.3	78.0	93.2	88.4	92.4
		**C57BL/6 mice**						**C3H mice**					
**Myeloblast**			NaCl/CTX vs. rBCG/CTX *p* = 0.001					NaCl/CTX vs. BCG/CTX *p* = 0.002					
**Promyelocyte**			NaCl/CTX vs. BCG/CTX *p* = 0.003					NaCl/CTX vs. rBCG/CTX *p* = 0.04					
			NaCl/CTX vs. rBCG/CTX *p* = 0.02										
**Metamyelocyte**			NaCl/CTX vs. BCG/CTX *p* = 0.02					NaCl/CTX vs. BCG/CTX *p* = 0.002					
			NaCl/CTX vs. rBCG/CTX *p* = 0.003										

## Data Availability

Not applicable.

## Supplementary Materials

The following are available online at https://www.mdpi.com/article/10.3390/vaccines10040615/s1: Figure S1: Forward (FSC) and side scatter (SSC) images of splenocytes isolated from the spleens of immunocompetent (A) and immunosuppressed (B) C57BL/6 mice. One representative image has been shown per group, Figure S2: Fluorescence minus one (FMO) controls of splenocytes stained with all of the fluorochromes (CD127 FITC, CD62L, PE-VIO770, CD44 V500, CD4 APC-CY7, CD8 APC) minus one of them. FMO1 control containing all fluorochromes except CD8 APC; CD4 APC-CY7 (FMO2); CD44 V500 (FMO3); CD62L PE-VIO770 (FMO4); and CD127 FITC (FMO5), Figure S3: Representative gating strategy to identify CD4^+^ and CD8^+^ T-cell subsets expressing markers for T_CM_ and T_EM_ in the murine spleens.
