# Supplementary material for: Mycobacterium bovis Wild-Type BCG or Recombinant BCG Secreting Murine IL-18 (rBCG/IL-18) Strains in Driving Immune Responses in Immunocompetent or Immunosuppressed Mice
Source: Vaccines (Basel). 2022 Apr 14;10(4):615. doi: 10.3390/vaccines10040615 (PMC9030902; doi:10.3390/vaccines10040615)
Supplement: Supplementary file 1 [file vaccines-10-00615-s001.zip › vaccines-1636060-supplementary.pdf]

Supplementary Materials

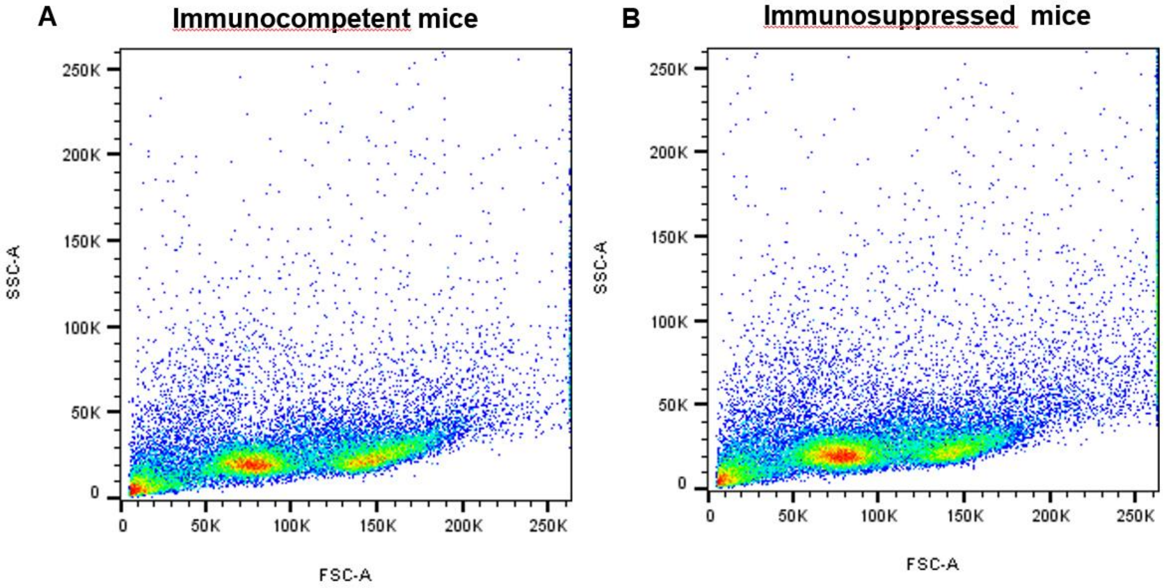

Figure S1. Forward (FSC) and side scatter (SSC) images of splenocytes isolated from the spleens of immunocompetent (**A**) and immunosuppressed (**B**) C57BL/6 mice. One representative image has been shown per group.

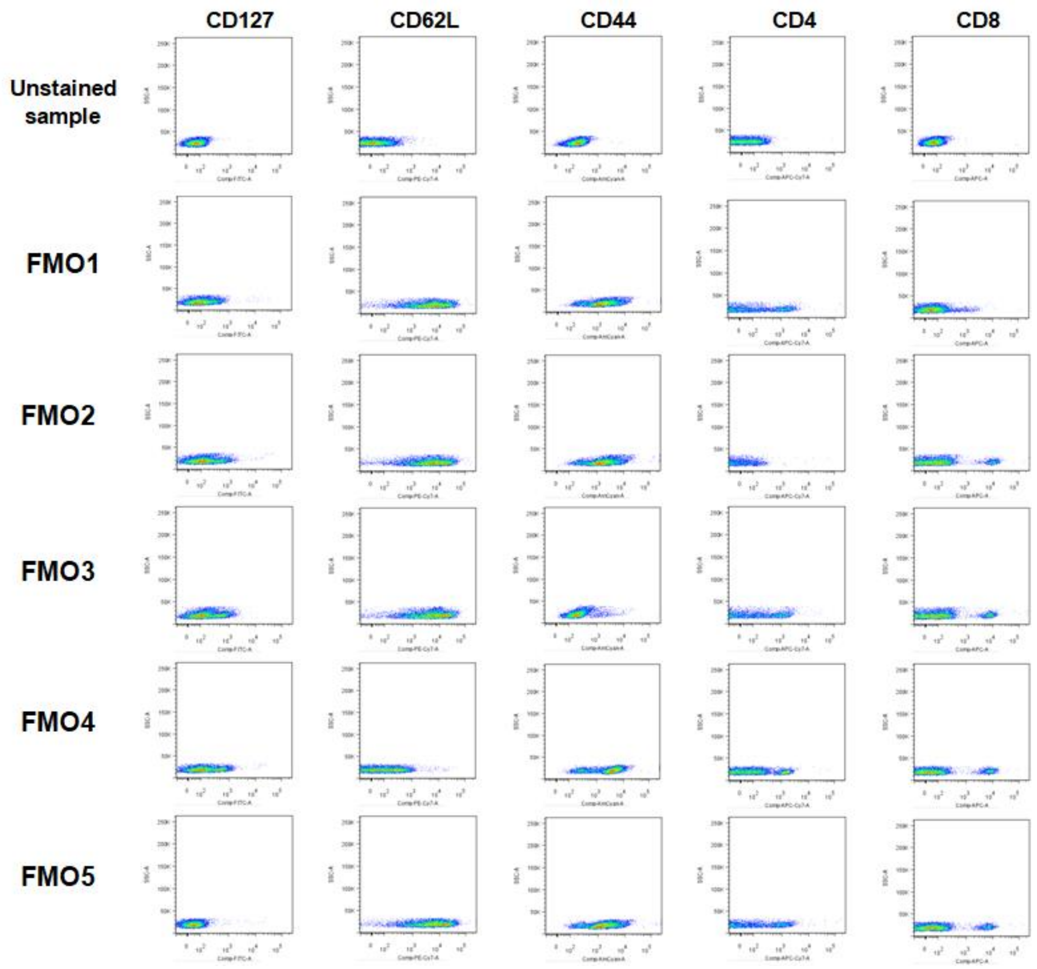

Figure S2. Fluorescence minus one (FMO) controls of splenocytes stained with all of the fluorochromes (CD127 FITC, CD62L, PE-VIO770, CD44 V500, CD4 APC-CY7, CD8 APC) minus one of them. FMO1 control containing all fluorochromes except CD8 APC; CD4 APC-CY7 (FMO2); CD44 V500 (FMO3); CD62L PE-VIO770 (FMO4); and CD127 FITC (FMO5).

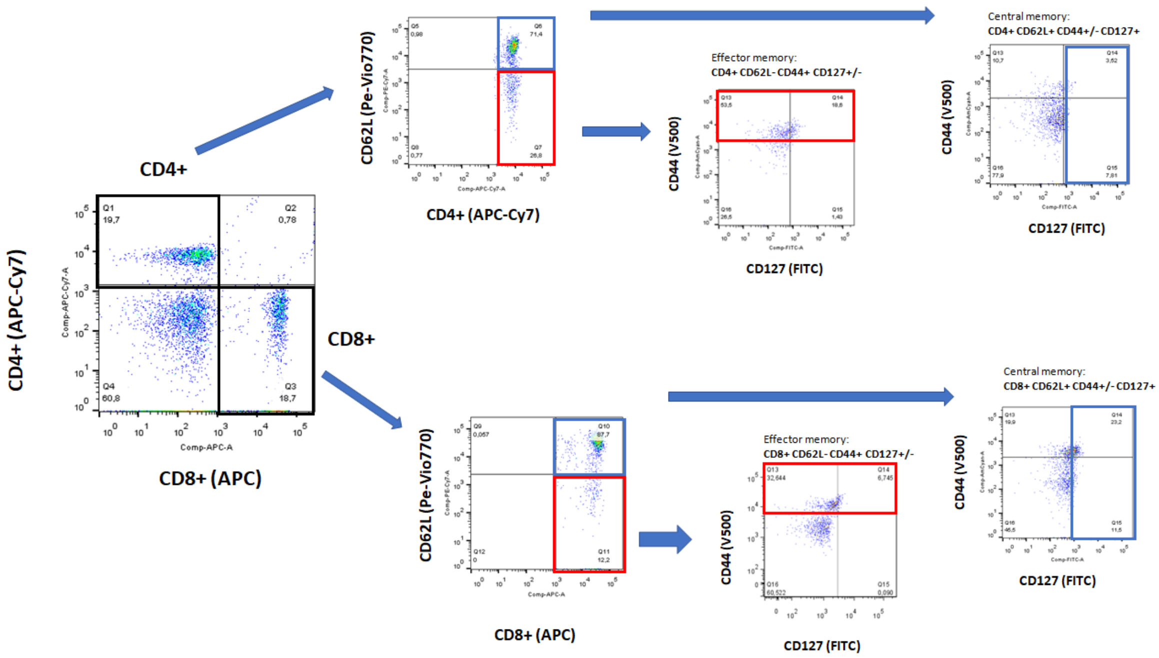

Figure S3. Representative gating strategy to identify CD4+ and CD8+ T-cell subsets expressing markers for TCM and TEM in the murine spleens.
